# Enhancing Free-Living Fall Risk Assessment: Contextualizing Mobility Based IMU Data

**DOI:** 10.3390/s23020891

**Published:** 2023-01-12

**Authors:** Jason Moore, Samuel Stuart, Peter McMeekin, Richard Walker, Yunus Celik, Matthew Pointon, Alan Godfrey

**Affiliations:** 1 Department of Computer and Information Sciences, Northumbria University, Newcastle upon Tyne NE1 8ST, UK; 2 Department of Sport, Exercise and Rehabilitation, Northumbria University, Newcastle upon Tyne NE1 8ST, UK; 3 Northumbria Healthcare NHS Foundation Trust, Newcastle upon Tyne NE1 8ST, UK; 4 Department of Nursing and Midwifery, Northumbria University, Newcastle upon Tyne NE1 8ST, UK

**Keywords:** gait, wearables, free-living, computer vision, terrain, environment

## Abstract

Fall risk assessment needs contemporary approaches based on habitual data. Currently, inertial measurement unit (IMU)-based wearables are used to inform free-living spatio-temporal gait characteristics to inform mobility assessment. Typically, a fluctuation of those characteristics will infer an increased fall risk. However, current approaches with IMUs alone remain limited, as there are no contextual data to comprehensively determine if underlying mechanistic (intrinsic) or environmental (extrinsic) factors impact mobility and, therefore, fall risk. Here, a case study is used to explore and discuss how contemporary video-based wearables could be used to supplement arising mobility-based IMU gait data to better inform habitual fall risk assessment. A single stroke survivor was recruited, and he conducted a series of mobility tasks in a lab and beyond while wearing video-based glasses and a single IMU. The latter generated topical gait characteristics that were discussed according to current research practices. Although current IMU-based approaches are beginning to provide habitual data, they remain limited. Given the plethora of extrinsic factors that may influence mobility-based gait, there is a need to corroborate IMUs with video data to comprehensively inform fall risk assessment. Use of artificial intelligence (AI)-based computer vision approaches could drastically aid the processing of video data in a timely and ethical manner. Many off-the-shelf AI tools exist to aid this current need and provide a means to automate contextual analysis to better inform mobility from IMU gait data for an individualized and contemporary approach to habitual fall risk assessment.

## 1. Introduction

Neurological conditions impair cognition and mobility, which can lead to an increased risk of falls. Approximately 60% of people with a neurological condition such as Parkinson’s disease report at least one fall per year, with 39% reporting recurrent falls [1,2]. Additionally, stroke survivors fall often in the early period after discharge; >70% experiencing at least one fall within 6 months [3]. To date, a current limitation of any fall risk assessment strategy is that it is not tracked in a real-world environment (i.e., remote/free-living/habitual (accessed on 17 December 2022), and there are no objective markers to understand this on an individual level. However, one contemporary mode of investigation is the use of wearable technologies to personalise fall risk assessment for use within habitual settings [4]. Current trends in wearable research align to the nuanced insights from a detailed mobility analysis to tailor fall prevention strategies to the individual [5].

**Figure 1:**
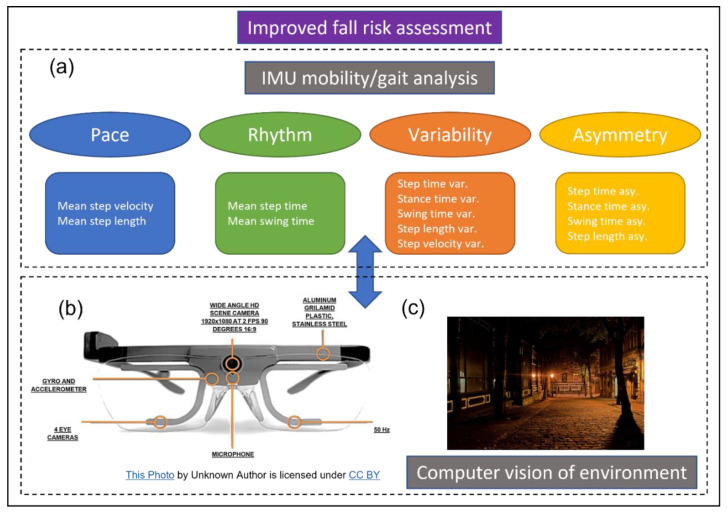
The proposal for a more informed and improved fall risk assessment. (**a**) IMU mobility-based gait data are useful but lack contextual information. (**b**) As technology in wearable glasses becomes more advanced, they could be a viable option to provide more routine video capture to augment (and better inform) IMU data captured for mobility-based gait analysis. (**c**) Video data could provide absolute clarity on environment and why e.g., high (step time) variability may occur such as on uneven pavement in a poorly lit setting.

Specifically, wearable inertial measurement units (IMU) consist of an accelerometer and gyroscope capable of high resolution (e.g., 100 Hz) data capture and are at the forefront of capturing clinically relevant habitual mobility-based data to better inform fall risk assessment [6,7]. In addition to providing high-resolution data, these wearables are capable of big data capture due to the typical recording time periods (e.g., 7 days) and offer extreme portability (e.g., worn on the person, under clothing). Typically, wearable IMUs can robustly quantify spatio-temporal gait characteristics (Figure 1a), providing valuable mobility data to provide some insight to an individual’s habitual fall risk [8]. For example, unstable/unsafe alternations in mobility manifested through high/abnormal gait characteristics can be represented by an increased step time variability [9]. If that is quantified in a supervised setting (e.g., clinic/laboratory) under the examination of a trained clinician, well informed assumptions and suggestions can be made to help reduce gait variability, tempering unsafe mobility alterations to reduced falls risk [10]. Although the same mobility-based gait characteristic can be quantified beyond the lab, the current limitation is that there is no contextual information to derive an informed assumption and more appropriate suggestion(s) to help reduce habitual fall risk [11]. For example, IMU-based high step time variability captured beyond the lab in a stroke survivor could be due to an intrinsic (e.g., disruption of neural pathways in the motor cortex) or extrinsic factor (e.g., uneven terrain). There is a need to augment habitual wearable IMU-based mobility assessment to better inform free-living fall risk.

Studies have begun to address this limitation using wearable cameras [12]. To date, methodologies have included utilising generic commercial cameras (e.g., GoPro^®^, https://gopro.com/en/gb (accessed on 17 December 2022)) mounted at the waist and aimed downwards at the feet, which is not only less ergonomic and more obtrusive for the participant, but it also fails to provide wider environmental context. Other studies have utilised cameras mounted at the chest to provide wider context to the scenes (e.g., identification of upcoming hazards or obstacles); however, the limitation of having cumbersome and unnatural technology attached to a participant remains. Compounding the issues arising from the previous methodologies are the number of ethical concerns and general procedural problems given that a researcher is required to manually review the video data. Ethically, having a researcher review and label hours’ worth of video showing a participant’s general view is problematic, as it can lead to scenarios such as a participant not turning the camera off during a private/sensitive moment like going to the toilet or having faces captured within the video image without consent [13]. Additionally, the process of manually reviewing a video is cumbersome, with review of an hour-long video typically taking many minutes/hours longer than the actual recording time [14].

Those challenges enable opportunities for automation [14], using computer vision-based artificial intelligence (AI) algorithms that can synchronise both IMU and wearable video data whilst also annotating/labelling the context of the environments (e.g., indoor/outdoor, type of terrain). Therefore, this case study explores and discusses how wearable video-based data capture can be used to better inform mobility-based fall risk within free-living environments. Additionally, this case study proposes a plausible computer vision processes for use on wearable video data to enable an automated approach for better IMU-based mobility analysis, leading to improved fall risk assessment. Here, this case study:

- Uses a contemporary and pragmatic video-based wearable to describe how context can better inform data from a lower back mounted IMU in fall risk assessment,
- Presents a suggested AI-based approach to automatically contextualise video data.

## 2. Materials and Methods

This preliminary investigation presents a single participant report to explore the role of a wearable camera to supplement mobility-based gait data captured by an IMU from within and beyond the laboratory. Pragmatic wearable technologies were worn by a participant, providing a detailed single case study to explore why use of video data is important, i.e., to better inform free-living mobility and habitual fall risk assessment. Here, IMU and video data were continuously captured from scripted mobility tasks within a university setting.

### 2.1. Participant

Ethical consent was granted by the Northumbria University Research Ethics Committee (REF: 21603, approval date: 11 February 2020). The single subject participant gave informed written consent before participating in this study. Testing took place at the Clinical Gait Laboratory and wider Coach Lane Campus, Northumbria University, Newcastle upon Tyne. The participant tested was an older adult (male, 72 years) stroke survivor who presented with hemiplegia, i.e., slight reduced clarity of movement on his left side.

### 2.2. Protocol

The participant conducted a series of (single and dual task) mobility tasks, consisting of both indoor and outdoor walking. The participant walked unaided around a range of different environments throughout the campus on a range of terrains. Firstly, the participant performed two 2-min (min) walks around a circuit in a controlled gait lab, followed by a long continuous walk around the university campus with a researcher. During all walks, the participant wore head-mounted camera-based glasses and an IMU mounted on the lower back (5th lumbar vertebrae, L5). For the purposes of this work, 30 s periods of video and corresponding IMU data from walks on contrasting terrain types were used only.

For lab-based dual tasking, the participant was tested at his maximum forward digit span, which was determined while sitting, and defined as the longest digit span the participant could successfully recall in two of three attempts. Digit strings consisted of pre-ordered numbers read aloud by a researcher. For the dual-task condition, the participant listened to digit strings while walking (for 2 min) and repeated them back.

### 2.3. Wearable Camera

Typically, wearable technologies are designed to integrate into the wearer’s daily life. That is routinely evidenced through wearable fitness trackers [15]. Yet, there are many sensing technologies that must be reimagined for routine adoption in daily life to overcome any stigma associated with their use [16]. One such technology is the camera which could provide a range of useful real-world data pertaining to fall risk e.g., absolute clarity on the effect of terrain to negatively impact mobility. Contemporary options exist such that video-based sensing could be routinely integrated into everyday life by continuous use through camera-based glasses (Figure 1). For example, the Invisible device proposed by Pupil Labs (https://pupil-labs.com/products/invisible/ (accessed on 19 December 2022)). Here, this study utilises similar technology to propose a contemporary approach for wearable camera deployment.

The glasses used in this preliminary study were the Tobii Pro 2 (Tobii, https://corporate.tobii.com/ (accessed on 19 December 2022)), which is capable of capturing high definition video at 1920 × 1080 pixels (px) at 30 frames/second (fps). Specifically, the glasses comprise two cameras: one facing outward from the frame to capture the environment where the wearer is looking, as well as an inward-facing infrared camera (with accompanying infrared light emitting diodes) to capture eye position. Accordingly, these glasses provide an overlaid crosshair to display eye location on the resulting (outward facing) video. This resulting crosshair provides exact clarity regarding the location of the participant’s visual focus throughout the entire video, allowing for the correlation of events with the participants gaze at the time. For the purposes of this preliminary study, only data from the outward-facing camera will be explored (Figure 1).

### 2.4. Wearable IMU

The field of mobility-based gait analysis is readily adopting the use of IMUs due to their more affordable cost (compared to e.g., instrumented walkways) and unobtrusiveness while capturing data in a range of environments [17]. Currently, there are a plethora of IMU devices used for mobility-based gait analysis [18]. Here, the AX6 (Axivity, https://axivity.com/ (accessed on 19 December 2022)) was used and secured to the participant at L5 via a waist strap. Accelerometer signals were recorded at a sampling frequency of 100 Hertz (Hz), and IMU configured (16-bit resolution, ±8 g) prior to data collection. Data were downloaded to a laptop upon completion of recording and analysed via validated algorithms, below.

### 2.5. IMU Algorithms and Mobility-Based Gait Characteristics

There are many IMU-based algorithms for mobility-based gait assessment specifically for use in neurological cohorts [19]. Generally, the field has aligned to the use of a single IMU on L5 and use of 14 spatio-temporal characteristics which map to four mobility-based gait domains [20], as shown in Figure 1. Many other characteristics can be generated, such as those from the frequency or time–frequency domain [11] but those remain limited for routine use in daily clinical practice due to inadequate clarity on how they could inform rehabilitation strategies [8].

**Figure 2:**
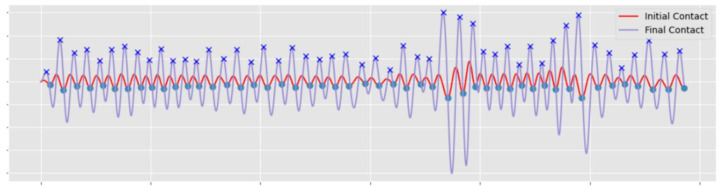
Example wavelet outputs from the bouts of walking captured during level ground asphalt walking phase with minima and maxima (peaks i.e., ICs and FCs) identified from CWT-based signals.

Here, IMU data were segmented from the continuous stream of data captured during the duration of the mobility tasks by a previously described and validated approach [21]. In brief, the combined tri-axial inertial data and vertical acceleration (*a_v_*) were used to identify possible moving and upright periods, respectively. Subsequently, those periods of interest were subjected to further investigation, whereby the identification of consecutive initial contact (IC) and final contact (FC) gait cycle events suggested periods of mobility-based gait activity and the required spatio-temporal characteristics, as shown in Figure 1. The following briefly describes IC/FC detection and mobility-based gait characteristics [22,23]:

1. A continuous wavelet transform (CWT) estimated IC and FC time events from *a_v_*. First, *a_v_* was integrated and then differentiated using a Gaussian CWT, ICs were identified as the times of the minima. The differentiated signal underwent a further CWT differentiation from which FC’s were identified as the times of the maxima, Figure 2. From the sequence of IC/FC events produced within a gait cycle temporal characteristics (e.g., step time) were generated.
2. IC and FC values were used to calculate an array of times for: (i) step (time from IC_Right_ to IC_Left_ to IC_Right_, etc.), (ii) stance (time between IC_Left_ and FC_Left_, then IC_Right_ and FC_Right_, etc.) and swing (time between FC_Left_ and IC_Left_ then FC_Right_ and IC_Right_, etc.), [24].
3. The spatial characteristic of step length was estimated from the up/down movement of participants centre of mass (CoM), close to L5. Movement in the vertical direction follows a circular trajectory and the changes in CoM height can be calculated (double integration of *a_v_*) to produce step length from the inverted pendulum model [23]. A combination of step time and step length produce step velocity [24].

### 2.6. Analysis

IMU-based data with bouts of walking such as navigating stairs or curbs were manually extracted and contrasted with other bouts by comparing the mobility-based gait characteristics. Firstly, the array of spatio-temporal characteristics were examined (Section 3.1). Then, to adopt a contemporary mobility assessment approach, the mean, variability and asymmetry of gait data were presented and discussed (Figure 1a). Variability was calculated as the standard deviation across all detected steps in every 30 s bout, while asymmetry was calculated as the absolute values of the difference between means of every other step (i.e., left, and right) within the same 30 s bout.

## 3. Results and Discussion

The purpose of this case study was to propose and explore the approach of a contemporary wearable camera to supplement IMU-based mobility-based assessment to provide more insight for arising gait characteristics. It is proposed that the approach can provide a method for better free-living fall risk assessment.

Here, this paper presents a breakdown of extracted spatio-temporal mobility-based gait characteristics obtained from an IMU on the lower back, along with contextual environmental data obtained from concurrent videos gathered by wearable glasses. When analysed, it is proposed that video data can provide explanations to account for variations in the extracted mobility-based IMU gait characteristics, dependent on environment (indoor/outdoor) and the terrain the participant was navigating. Data are presented and discussed in the following sections by investigating mobility-based gait outcomes and CWT signals with contextualisation from use of video data.

### 3.1. IMU Mobility-Based Gait Data: Current Limitations and Future Considerations

**Figure 3:**
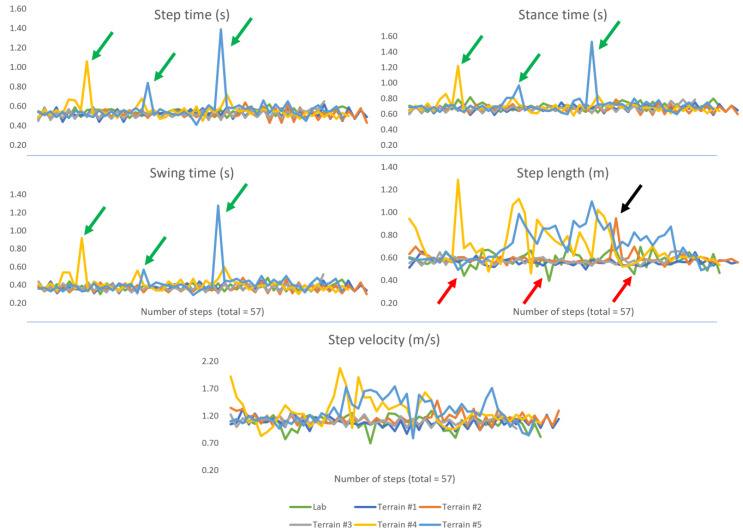
Spatio-temporal mobility-based gait characteristics from 30 s (≈57 steps) bouts of walking across the numerous terrain types. Here, we observed the greatest anomalies in the characteristics on terrains #4 (stair ascent, yellow) and #5 (stair descent, light blue). Typically, the remaining terrains present characteristics in what may appear to be normal fluctuations. Of note, the step length and step velocity in the lab bout (green) show some altered fluctuations and may be attributed to the protocol i.e., a walk in a looped (non-linear) circuit.

**Figure 4:**
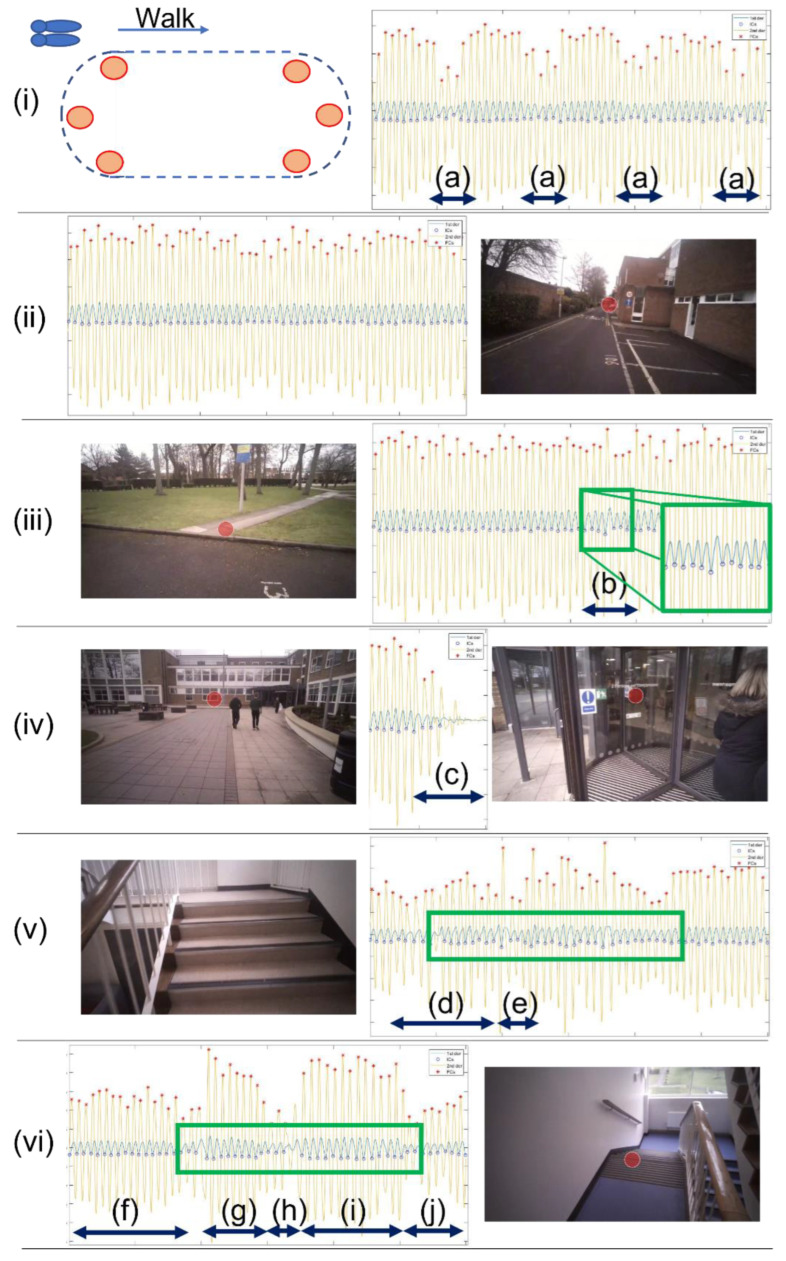
Walking bout IMU processed and video data. (**i**) 30 s (from continuous 2 min loop) within a lab, (a) denoting the turns at the end of the track, (**ii**) level walking on asphalt with no clear abnormality in the IMU data but walking was in a linear path (i.e., straight line), (**iii**) walk from asphalt to paving and a step identified by the participant (red dot), with a possible anomaly in IMU-based data from visual observation denoted by (b and green squares), which roughly equates to the time of a single step from asphalt to paving, (**iv**) walking on paving and (c) at the latter stages of the 30 s walk as the participant stops before entering a revolving door, (**v**) stair ascent, noticeable changes in the CWT-based data as the participant walks up steps (d) and turns left (e) on the landing, and (**vi**) noticeable changes in the CWT-based data from level walking (f), stair descent (g), short steps to turn to next flight (h), stair descent (i) and level walking (j). The green rectangle in (**v**,**vi**) highlighting the similar fluctuations, as observed in (**iii**), due to stepping.

Overall, when navigating level terrain (lab, #1 asphalt, #2 asphalt + paving and #3 paving alone), little to no observable difference can be identified across the range of extracted mobility-based temporal IMU gait characteristics within 30 s bouts (≈57 steps), as shown in Figure 3. Yet, some anomalies/peaks are observed in step length, as denoted by the red and black arrows in Figure 3. Current approaches of free-living mobility-based assessment based on IMU data alone fail to understand why those anomalies could exist and what causes the participant to have reduced and/or inflated step lengths. In short, there is a failure to comprehensively understand if (i) underlying intrinsic or (ii) the environment/terrains and associated extrinsic factors are influencing the participant’s mobility-based gait. The use of wearable video data enables a more insightful examination and understanding of the IMU-based characteristics. By augmenting mobility-based IMU gait characteristics with video data, the impact of extrinsic (and not necessarily intrinsic) factors on mobility emerge:

1. The lab protocol consisted of a 2-min walk in a loop which, although it is a scripted task, is useful to examine. Step length characteristics show reduced measurements (Figure 3, red arrows) at periodical moments, which are explained by the reduced stepping distance as the participant rounded the ends of the curved path of the circuit (Figure 4i). Though the step length (inverted pendulum) algorithm is designed for straight level walking, it is sensitive enough to detect shorter step lengths during obtuse angled curved walking i.e., gradual turns compared to acute turns. Accordingly, the added context (video) is useful for understanding the reduced stepping length, which could help provide a better understanding of natural intrinsic considerations to extrinsic factors, such as purposefully adjusting direction of travel to round obstacles or other pedestrians. This is a simple example within the lab but the usefulness of examining step length is also evidenced beyond controlled conditions.
2. In Figure 3, step length is the one obvious anomaly from terrain #2 (black arrow), asphalt to paving (Figure 4iii). Interestingly, the increased step length (anomaly) occurred at the approximate time it was observed (from corresponding video), when the participant performed a step and transitioned from asphalt onto paving. This matches the corresponding CWT derived IMU-based signals of Figure 4iii, where there is a very subtle change in the 1st derivation of *a_v_* (Figure 4iii-b). Generally, it could be that occasional anomalies in step length data equate to routine adjustments in the daily mobility of a participant undertaking a single step transitioning from one terrain to another. Equally, it could be a natural avoidance of an obstacle on the ground (i.e., stepping over or beyond) due to natural intrinsic/instinctive reactions. Accordingly, it may be important to examine those step length anomalies arising from IMUs in accordance with video data to better understand the natural abilities of the participant to adjust and manage naturally occurring obstacles/hazards. The approach may help future areas of research aiming to refine IMU-based mobility-based gait data from continuous monitoring (e.g., 7-days) for targeted areas of investigation in fall risk assessment.

When more challenging terrains were identified (by video) and examined (i.e., #4 stair ascent and #5 descent) more significant fluctuations/changes are observed in all gait data, Figure 3. On those terrains, the (environmental) complexity produces extrinsic factors (e.g., numerous repeated steps in an upward or downward trajectory) resulting in some clear anomalies in temporal characteristics (step, stance, and swing times), Figure 3 (green arrows). Although the underlying CWT algorithm is not specifically designed to quantify mobility-based gait from stair ascent or descent, it generally appears to function no differently compared to temporal IMU data captured during level walking, giving the appearance that its end mobility-based gait characteristics are valid (ignoring the occasional anomaly). Perhaps this is due to its fundamental principle of identifying the low-frequency accelerations of the pelvic/L5 region, which may be similar during stair ambulation compared to level walking. Indeed, other research has shown that net moments of force (albeit) at the hip are the same for stair walking and level walking [25]. Additionally, Table 1 presents mean temporal data and there appears to be no major observable difference across all 5 terrains (other than swing time on terrain #2). Perhaps, the stairs (and possibly the use of visual cues such as black stripes to readily identify each step) may regulate/cue the participant, meaning temporal data on these terrains (#4 and #5) appear equal/similar from those obtained during level ground walking when, in fact, they should (fundamentally) not be treated as equal i.e., not included in the same analysis due to biomechanical differences needed for mobility on such contrasting terrains (i.e., level vs. stairs).

Discrepancies between level and stair terrains become more observable when utilising mean spatio-temporal as well as all variability and asymmetry characteristics, as shown in Table 1. Specifically, step length and velocity have more obvious anomalies on these terrains compared to others i.e., highly fluctuating characteristics compared to their equivalent data on level ground, as shown in Figure 3. Here, these CoM-derived characteristics are dependent on the inverted pendulum model, designed for use on linear and flat level walking only [23]. Accordingly, use of the model for step length and velocity characteristics is clearly not fit for purpose on terrains like stairs, and so their resulting values are (i) fundamentally incorrect and therefore (ii) misleading in terms of the participant’s true mobility-based gait. However, current approaches that do not use video data have no absolute clarity to determine why the discrepancy in spatio-temporal, variability and asymmetry mobility-based gait characteristics may occur from one mobility bout to another (i.e., from lab and terrains #1 to #3 compared to #4 and #5). Although, if the research focus is to broadly examine fall risk, then perhaps current IMU-based mobility-based gait data from L5 may remain a useful proxy (Section 3.3).

Increased variability within step-to-step events is often cited as the most impactful predictor of fall risk [26,27], and use of a single IMU on L5 is a common mechanism to quantify free-living gait. Table 1 shows variability characteristics, which display an interesting pattern. Although there may be no true IC/FC event from stair ambulation due to foot placement, the CWT algorithm produces (what seem like) plausible temporal characteristics due to the proxy nature of the algorithm (highlighted in previous section). Specifically, the algorithm infers times derived from underlying frequency variations due to IC/FC events [22] that are distal (feet) to the IMU site of attachment (L5) [6]. Regardless of the lack of fundamental accuracies of IC/FC in gait cycle, all quantified variability characteristics show changes from level to stair terrains. Broadly and perhaps expectedly, there seems to be a linear relationship between increasing terrain complexity and temporal gait characteristic variability (SD) from the lab to outdoor level terrain (asphalt, asphalt + paving and paving), to stairs (Table 1).

Interestingly, a linear relationship is not evident from variability and asymmetry spatio-temporal gait from the lab to outdoor level terrain (Table 1). It can be rationalised that the researcher impacted the participant’s normal walking style. This can be evidenced from video analysis where it was observed that the participant occasionally gazed at the researcher while engaging in conversation causing occasional moments of hesitancy and therefore, an altered stepping pattern. Here, the use of video data is particularly useful to help guide IMU characteristics in comparison to lab-based data. Additionally, variability greatly increased for stair ambulation (Table 1) due to the obvious biomechanical differences required and arising impact on the inverted pendulum model used to quantify step length and velocity. Again, use of video data clearly identified stair ambulation.

**Figure 5:**
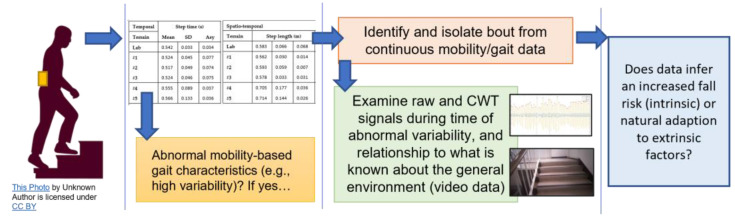
Left to right, although common algorithms for spatio-temporal processing for data collect on L5 are not specifically designed for e.g., stair ambulation, they may still be useful tools to help inform fall risk when used with video data for a more rounded mobility assessment.

Although the use of the CWT and inverted pendulum algorithms may not be specifically designed for some terrains (Table 1) and are unlikely to produce valid data when considered within the gait cycle on those terrains, they seem to produce data that could be generally useful to broadly identify increased complexity within common mobility tasks. For example, the identification of time periods within continuous IMU data for when a participant data is abnormal could be useful to segment video data and determine if the cause is due to intrinsic or extrinsic factors, as shown in Figure 5. Accordingly, that approach may help streamline IMU and video processing while helping to better investigate fall risk that continues to be limited by assumptions needing to be made of intrinsic or extrinsic factors.

### 3.2. Other Observations

Based on current approaches (single IMU on L5), there is no absolute clarity to determine whether intrinsic or extrinsic factors are impacting mobility-based gait from the quantification of characteristics, as shown in Figure 4. Although data may remain somewhat useful to broadly infer assumptions on fall risk (e.g., higher gait variability in habitual settings compared to a lab), the continued use of a device at that anatomical location remains limited if the research focus is on the fundamentals of gait cycles alone, as no targeted interventions can be developed to suit the individual needs of a participant. Furthermore, there are many other natural occurring extrinsic factors that impact mobility-based gait and increased fall risk. For example, pausing/stopping at a doorway (Figure 4iv), which has been shown to produce notable mechanistic impact on specific regions of the brain which may encode environmental cues to adapt mobility-based gait [28].

Doorways are well documented in Parkinson’s research, eliciting various stimuli that evoke abnormal gait characteristics, resulting in episodes of freezing [29]. In our example (Figure 4iv), the CWT signals begin to show a reduction in amplitude as the participant slows to firstly allow another pedestrian through the door before he advances. That routine event may occur often during a person’s community-based ambulation, but is important to isolate, to determine habitual mobility-based gait characteristics during these specific and challenging tasks which may seem to produce abnormal values indicative of increased falls risk. This is important, as arising characteristics are proposed as useful to explore underlying mechanisms to aid safe and effective mobility and therefore, reduce fall risk.

### 3.3. A Conceptual Model with Context

Although a general examination of mobility-based characteristics (Table 1) may be useful for an initial fall risk assessment with current IMU approaches, stratifying characteristics according to conceptual models (Figure 1) may be more helpful to better understand discrete cognitive functions [30] and the effectiveness of targeted interventions [31]. Table 2 stratifies all mobility-based gait characteristics according to a free-living conceptual model [20], the use of which may enable more sensitive methods to better identify the use of a targeted intervention [32], particularly when the associated environmental context is known.

Here, mobility-based gait characteristics are presented under single (quiet walking) and dual task (number recall) to compare with data from beyond the lab, where naturally occurring stimuli may often arise due to the uncontrolled environment, e.g., as noted, our participant engaged in conversation and glancing at the researcher. As might be expected from the lab data, in comparison to the single task, the dual task condition reduced the participants pace and increased their rhythm and variability, as shown in Table 2. No obvious pattern is evident when comparing lab-based single and dual task data to outdoor terrain. However, the use of video data show terrains #1 to #3 were on level ground, with walks conducted in a linear manner (i.e., no curved deviations in comparison to the lab). In contrast, video data readily identified stairs (#4 and #5) with higher pace, rhythm and variability characteristics. Again, those data cannot be considered as valid (Section 3.1), but merely as a proxy to indicate a period of time within mobility-based IMU gait data that should be used in conjunction with video data to thoroughly assess fall risk.

### 3.4. Further Context and Next Steps

As broadly demonstrated, the use of wearable cameras could enable further insights beyond environment and terrain, additional daily extrinsic factors such as entering doorways, which may impact mobility-based gait and increase fall risk. Additionally, as described, our participant was accompanied by a researcher during his walk beyond the lab. The head-mounted video-based glasses showed that the participant turned his head many times during his walk to face the researcher while undertaking a conversation. If future studies are to continue using IMUs for mobility-based gait to inform fall risk, then accompanying contextual information needs to be gathered as well, such as if the participant is, e.g., walking and talking with another person. Accordingly, due to any underlying functional physical limitation (e.g., hemiplegia) and an additional (distracting, dual tasking) cognitive task (s), the mobility-based IMU gait may be sufficiently altered to robustly inform fall risk. Indeed, the effect of distractions on situational awareness and the subsequent (negative) impact on gait is evidenced elsewhere [33,34,35], playing an important role in the control of mobility and, therefore, fall risk.

For this exploratory and preliminary case study examination, a manual approach was used to segment and assess video data. Here, this process was not overly time consuming due to the scripted nature of the protocol and use of validated IMU-based algorithms to segment associated mobility-based gait data. However, for pragmatic use of video data to augment IMU-based mobility data for better fall risk assessment, a more automated approach would be preferred. An automated, artificial intelligence (AI) approach would expedite the process while also potentially protecting the privacy of the participant. Next, one possible AI approach is presented.

### 3.5. Computer Vision-Based Algorithm

There are many approaches/algorithms that could be used to automatically analyse video arising from a wearable camera [36,37]. Here, the purpose is not to verify or validate any one algorithm, but rather to showcase the use of a computing vision approach to undertake the proposed work outlined. Typically, the need for AI computer vision-based algorithms is to classify everyday environments, i.e., whether a participant is indoor/outdoor and on what terrain they are walking. Approaches typically involve CNNs (convolutional neural networks) developed using a python-based deep learning library (e.g., PyTorch/TensorFlow) and either involve mobile waist-mounted cameras aimed directly at a participant’s feet [38] or utilise further pre-processing steps on the image stemming from head-mounted video glasses to extract and classify floor location [39]. For more nuanced classification of external factors affecting gait, object detection algorithms can be implemented.

Given the much wider application of generalised object detection algorithms, more off the shelf algorithms are widely available, such as the you only look once (YOLO) series of algorithms [40,41,42] or generalised object segmentation algorithms [43,44]. Typically, these algorithms are available pretrained to identify and perform on widely available datasets such as ImageNet [45] or common objects in context (COCO) [46], allowing for the detection of objects such as chairs, cars, people and other common obstacles. This allows for the detection of obstacles within a participant’s path to be identified from field-of-view video data without the need for any large-scale model building or dataset labelling. One caveat to this approach is that, within fall risk assessment, areas of heightened interest would include instances in which a participant is navigating a curb, stairs or walking through doorways, none of which are possible using off-the-shelf algorithms trained on either ImageNet or COCO. To enable the algorithms to detect these instances, transfer learning may be the best approach, where an off-the-shelf algorithm is utilised, pretrained on either the ImageNet or COCO datasets, with the final classification layer mapping to the available classes replaced by a custom defined layer mapping to our required classes [47,48,49] (curbs, doorways, people, stairs, etc.) that are identified as impactful in terms of their extrinsic effect on gait. While this would require more effort on the part of researchers, the outcomes would be orders of magnitude more impactful on providing context to the correlated signals stemming from the IMU devices.

One key element of any contextual analysis identified from our preliminary examination was the distractor of the participant talking to researchers while walking. That action caused the participant to turn their head and focus on the researcher, which may have been a possible cause of higher gait variability within the mobility-based IMU gait characteristics and, without context, could be misunderstood as an intrinsic factor. Utilising the aforementioned object-detection AI algorithms, identification of people and, by extension, faces would allow for this context to be concatenated to the signal and mobility-based gait characteristics, providing the context that the participant was talking while walking. Furthermore, by identifying an individual in that context, the same AI could blur the face of the individual to protect their identity [50,51], upholding ethical concerns.

While the classification of currently navigated terrain is an important stepping stone to achieving complete environmental contextualisation, future work entails the implementation of further computer vision algorithms to extract potential hazards/obstacles within view in conjunction with wearable eye tracking devices to better understand how participants navigate their environments, with specific focus on detailing any obstacles discovered within a participant’s path and whether or not the participant had seen that obstacle by correlating eye location with the obstacle’s position within frame.

### 3.6. Video-Based Challenges

A key challenge with the proposed computer vision approach is the quality of camera provided by the wearable eye-tracking glasses. Given the micro size requirements of the camera to fit ergonomically within the headset, the quality of output video can be somewhat degraded. This is especially noticeable within poorly lit environments. However, as with all hardware developments, as time moves forward, expectations for the quality of the camera to increase are a given, allowing for the same algorithm to be applied, leading to more robust accuracies.

### 3.7. Ethical AI

There is a plethora of ethical considerations that must be considered when dealing with playback of wearable camera footage taken within free-living environments across a prolonged period. With the usage of automated AI-based methods to handle processing of the video itself, many of the previous ethical concerns are alleviated. However, ethical considerations of the usage and design of the AI itself must be considered. Typically, ethical AI represents topics such as preventing bias in AI algorithms; however, within this studies context, ethical AI represents removing sensitive information and scenarios from the viewable FPV-based scenes. This could be, for example, when a participant is viewing a bank statement while the cameras are active or during a sensitive event such as a participant using the toilet while forgetting to turn off the cameras. Future work within this area should involve the utilisation of computer vision-based object detection algorithms to begin to remove any sensitive environments or information (including faces, above) from the view of any researchers reviewing the data.

### 3.8. Enhanced Fall Risk Assessment

Falls can begin a cascade of financial, individual and social consequences [52]. At a time of an ageing population and increasing demands on health and social care providers, screening patients for fall risk and addressing risks has been shown to be cost-effective [52]. With the approach of providing automated context, a groundwork for the automated analysis of fall risk could be laid. Through use of an AI system, subjective opinions on whether areas of high gait variability are caused by intrinsic or extrinsic factors could be alleviated by providing absolute context to the terrain being traversed, along with whether the participant was, e.g., (i) within an indoor or outdoor environment, (ii) on a challenging terrain, (iii) with or without someone else or (iv) navigating a busy/complex environment. This offers the opportunity to better manage the risk of falling and to realise the benefits at an individual level.

## 4. Conclusions

This paper broadly discusses the current uses and limitations of a single IMU worn on the lower back, which was used within the field to inform fall risk from mobility-based gait. Here, a single participant stroke survivor was used to provide insightful data captured from various terrains within a lab and beyond. While some data derived from commonly used IMU-based algorithms seem somewhat useful for fall risk assessment, approaches remain limited due to a lack of comprehensive understanding of the context associated with mobility-based IMU gait characteristics. A thorough understanding of intrinsic and extrinsic factors may only be achieved by augmenting mobility-based IMU gait with a contemporary approach for video data capture, i.e., wearable video-based glasses. Many possible AI-based computing vision algorithms exist and should be investigated for their use to process contextual information ethically and efficiently from free-living environments to comprehensively inform fall risk assessment. The field of free-living fall risk assessment should consider the use of contemporary wearable video-based devices to better inform the current limited approach of IMU wearables only. Although the use of video has notable ethical concerns, many current automated AI-based approaches using computer vision may uphold participant anonymity and privacy. By supplementing mobility-based IMU gait data with video, a thorough and better understanding of fall risk from habitual/free-living environments can be determined.

## Figures and Tables

**Table 1:** Initial assessment of mobility/gait characteristics and terrain types over different 30 s bouts, no context.

Temporal	Step Time (s)			Stance Time (s)			Swing Time (s)		
Terrain	Mean	SD	Asy	Mean	SD	Asy	Mean	SD	Asy
**Lab**	0.542	0.033	0.034	0.701	0.046	0.053	0.381	0.038	0.053
**#1**	0.524	0.045	0.077	0.674	0.043	0.071	0.374	0.042	0.071
**#2**	0.517	0.049	0.074	0.670	0.046	0.071	0.670	0.044	0.071
**#3**	0.524	0.046	0.075	0.675	0.047	0.075	0.374	0.044	0.074
**#4**	0.555	0.089	0.037	0.699	0.092	0.042	0.412	0.096	0.045
**#5**	0.566	0.133	0.036	0.718	0.133	0.037	0.415	0.136	0.038
**Spatio-temporal**									
**Terrain**	**Step length (m)**			**Step velocity (m/s)**			**Mean:** average in a 30 s bout **SD:** standard deviation of times in a 30 s bout **Asy:** absolute difference between left and right (assumed as every other step) in a 30 s bout		
**Lab**	0.583	0.066	0.068	1.079	0.129	0.051			
**#1**	0.562	0.030	0.014	1.079	0.096	0.130			
**#2**	0.593	0.059	0.007	1.153	0.109	0.149			
**#3**	0.578	0.033	0.031	1.107	0.072	0.099			
**#4**	0.705	0.177	0.036	1.274	0.267	0.016			
**#5**	0.714	0.144	0.026	1.280	0.237	0.100			

**Table 2:** Mobility/gait characteristics and terrain types over 30 s walk, with context.

Mobility/Gait Domain	IMU Mobility/Gait Characteristics	Lab		Outdoor and Non-Laboratory Terrains				
		Single	Dual	#1	#2	#3	#4	#5
**Pace**	Mean step velocity (m/s)	1.079	0.967	1.079	1.153	1.107	1.274	1.280
	Mean step length (m)	0.583	0.548	0.562	0.593	0.578	0.705	0.714
**Rhythm**	Mean step time (s)	0.542	0.568	0.524	0.517	0.524	0.555	0.566
	Mean swing time (s)	0.381	0.414	0.374	0.366	0.374	0.412	0.415
**Variability**	Step time variability (s)	0.033	0.033	0.045	0.049	0.046	0.089	0.133
	Stance time variability (s)	0.046	0.045	0.043	0.046	0.047	0.092	0.133
	Swing time variability (s)	0.038	0.047	0.042	0.045	0.044	0.096	0.136
	Step length variability (s)	0.066	0.080	0.030	0.059	0.033	0.177	0.144
	Step velocity variability (s)	0.129	0.147	0.096	0.109	0.072	0.267	0.237
**Asymmetry**	Step time asymmetry (s)	0.034	0.027	0.077	0.074	0.075	0.037	0.036
	Stance time asymmetry (s)	0.053	0.049	0.071	0.071	0.075	0.042	0.037
	Swing time asymmetry (s)	0.053	0.048	0.071	0.071	0.074	0.045	0.038
	Step length asymmetry (s)	0.068	0.077	0.014	0.007	0.031	0.036	0.026
**Lab**—25 m circuit with obtuse curves at each end **#1**—outdoor: level ground, asphalt **#2**—outdoor: level ground asphalt, with one step onto paving (slightly uneven/irregular surface) **#3**—outdoor: paving but slightly uneven/irregular surface approaching a revolving door **#4**—indoor: level walking on vinyl with a slight right turn onto stair ascent (11 steps), turn left on landing to next stair ascent (11 steps), level walking with slight turn left through door onto carpet **#5**—indoor: level walking on vinyl in narrow corridor through door with slight turn right onto stair descent (8 steps), turn right on landing to next stair descent (13 steps), level walking with immediate turn left through door and into narrow corridor.								

## Data Availability

Data is available upon reasonable request.
